# A non-canonical Δ9-desaturase synthesizing palmitoleic acid identified in the thraustochytrid *Aurantiochytrium* sp. T66

**DOI:** 10.1007/s00253-021-11425-5

**Published:** 2021-07-22

**Authors:** E-Ming Rau, Inga Marie Aasen, Helga Ertesvåg

**Affiliations:** 1 Department of Biotechnology and Food Science, NTNU Norwegian University of Science and Technology, Trondheim, Norway; 2 Department of Biotechnology and Nanomedicine, SINTEF Industry, Trondheim, Norway

**Keywords:** Delta-9 desaturase, Palmitoleic acid, Aurantiochytrium, Thraustochytrids, Delta-12 desaturase, Fatty acid

## Abstract

**Abstract:**

Thraustochytrids are oleaginous marine eukaryotic microbes currently used to produce the essential omega-3 fatty acid docosahexaenoic acid (DHA, C22:6 n-3). To improve the production of this essential fatty acid by strain engineering, it is important to deeply understand how thraustochytrids synthesize fatty acids. While DHA is synthesized by a dedicated enzyme complex, other fatty acids are probably synthesized by the fatty acid synthase, followed by desaturases and elongases. Which unsaturated fatty acids are produced differs between different thraustochytrid genera and species; for example, *Aurantiochytrium* sp. T66, but not *Aurantiochytrium limacinum* SR21, synthesizes palmitoleic acid (C16:1 n-7) and vaccenic acid (C18:1 n-7). How strain T66 can produce these fatty acids has not been known, because BLAST analyses suggest that strain T66 does not encode any Δ9-desaturase-like enzyme. However, it does encode one Δ12-desaturase-like enzyme. In this study, the latter enzyme was expressed in *A. limacinum* SR21, and both C16:1 n-7 and C18:1 n-7 could be detected in the transgenic cells. Our results show that this desaturase, annotated T66Des9, is a Δ9-desaturase accepting C16:0 as a substrate. Phylogenetic studies indicate that the corresponding gene probably has evolved from a Δ12-desaturase-encoding gene. This possibility has not been reported earlier and is important to consider when one tries to deduce the potential a given organism has for producing unsaturated fatty acids based on its genome sequence alone.

**Key points:**

• *In thraustochytrids, automatic gene annotation does not always explain the fatty acids produced.*

• *T66Des9 is shown to synthesize palmitoleic acid (C16:1 n-7).*

• *T66des9 has probably evolved from Δ12-desaturase-encoding genes.*

**Supplementary Information:**

The online version contains supplementary material available at 10.1007/s00253-021-11425-5.

## Introduction

Thraustochytrids are heterotrophic marine eukaryotes known for the ability to accumulate high levels of the omega-3 fatty acid DHA (Aasen et al. 2016; Morabito et al. 2019). They belong to the *Stramenopiles* group and are divided into several genera such as *Schizochytrium*, *Aurantiochytrium*, and *Thraustochytrium* (Marchan et al. 2018). Omega-3 fatty acids, including DHA, are beneficial to human health, and some thraustochytrid strains are used for commercial DHA production (Barclay et al. 2010; Guo et al. 2018). Due to the increasing demand for sustainable sources of fatty acids (FAs), such as DHA (Ghasemi Fard et al. 2019; Sprague et al. 2016), knowledge about how thraustochytrids synthesize FAs is important to be able to improve potential production strains by metabolic engineering.

**Fig. 1:**
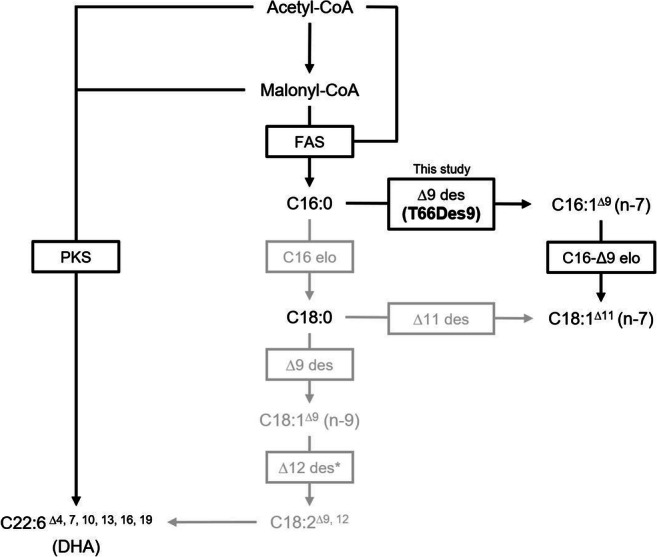
Possible pathways for biosynthesis of unsaturated fatty acids in *Aurantiochytrium* sp. T66. des, desaturase; elo, elongase; FAS, fatty acid synthase; PKS, PKS pathway. Enzymes not found encoded in the *Aurantiochytrium* sp. T66 genome and FAs never detected in this strain are written in gray. Single asterisk indicates the automatic annotation for T66Des9.

Polyunsaturated fatty acids (PUFAs) can be produced by two distinct pathways. Usually, PUFAs are synthesized from C16:0, the main product of the fatty acid synthase (FAS), by the desaturase-elongase (DE) pathway (Sun et al. 2020; Thelen and Ohlrogge 2002) (Fig. 1). The desaturases of the DE pathway are mixed function oxidases, and the reaction is coupled to an electron flow involving cytochrome b5 and cytochrome b5 reductase. However, in thraustochytrids producing DHA and in bacteria producing omega-3 fatty acids, PUFAs are mostly synthesized by an alternative pathway, using a dedicated polyketide synthase (PKS) which synthesizes, e.g., DHA directly from malonyl-CoA and acetyl-CoA without the need for oxygen for desaturation (Hauvermale et al. 2006; Metz et al. 2001). Although some PUFAs are synthesized by the DE pathway in some thraustochytrids, this pathway has not been shown to contribute to DHA production (Matsuda et al. 2012; Sakaguchi et al. 2012). Even though both the FAS-DE and the PKS pathways share precursors such as acetyl-CoA, malonyl-CoA, and NADPH, the regulation of the relative activities of these pathways is still mostly unknown.

Different thraustochytrid genera or strains have different FA compositions, potentially related to the presence or absence of particular enzymes. In a comparative study, it was reported that the amount of linoleic acid (C18:2 n-6), stearic acid (C18:0), and oleic acid (C18:1 n-9) in *Schizochytrium* sp. SEK210 and *Thraustochytrium aureum* ATCC 24473 were significantly higher than those in the tested *Aurantiochytrium* sp. (Nagano et al. 2011). Moreover, *T. aureum* has been found to produce n-6 PUFAs by the DE pathway (Matsuda et al. 2012), while FAs like C20:4 have not been detected in *Aurantiochytrium* sp. (Heggeset et al. 2019). In addition, monounsaturated FA palmitoleic acid (C16:1 n-7) and vaccenic acid (C18:1 n-7) have been detected in *Aurantiochytrium* sp. T66., but not in *Aurantiochytrium limacinum* SR21 (Jakobsen et al. 2008; Yokochi et al. 1998). As indicated in Fig. 1, C16:0 can be desaturated to C16:1 n-7 by Δ9-desaturases. C16:1 n-7 can then be elongated to C18:1 n-7 by an elongase accepting C16-Δ9 as substrate. Alternatively, C18:1 n-7 could be synthesized from C18:0 by a Δ11-desaturase.

The genome of *Aurantiochytrium* sp. T66 encodes seven putative desaturases, including gene T66002957.1, which exhibits high homology to Δ12-desaturases that catalyze the conversion of oleic acid (C18:1 n-9) to linoleic acid (C18:2 n-6) (Heggeset et al. 2019). However, no C18:2 n-6 or C18:1 n-9 were detected in the FA profile of *Aurantiochytrium* sp. T66 (Jakobsen et al. 2008). In addition, T66002957.1 was highly expressed at the nitrogen-limited lipid accumulation stage in *Aurantiochytrium* sp. T66 cells and that is when the two monounsaturated FAs are synthesized (Heggeset et al. 2019). Moreover, in *Thraustochytrium* sp. ATCC 26185, a putative desaturase with the identical amino acid sequence of the T66002957.1 encoded a protein that displayed no Δ12-desaturase activity when expressed heterologously in *Escherichia coli* (Meesapyodsuk and Qiu 2016), suggesting that the function of the enzyme could be different from that predicted by sequence similarity.

In this study, we explored the hypothesis that although the T66002957.1 encoded protein shares Δ12-desaturase sequence characteristics, it might possess Δ9-desaturase activity. Since *A. limacinum* SR21 has not been reported to produce C16:1 or C18:1 FAs, and was found not to encode a homologous protein, we expressed T66002957.1 in *A. limacinum* SR21. The transgenic strain synthesized both C16:1 n-7 and C18:1 n-7. T66002957.1 is therefore named *T66des9* and the corresponding protein T66Des9 in the rest of this paper.

## Materials and methods

### Strains and medium

*Aurantiochytrium limacinum* SR21 (ATCC® MYA-1381™) was cultured in GPYS [3% glucose, 0.6% peptone, 0.2% yeast extract, 50 mM sucrose, 1.8% ocean salt (Tropic Marin® Sea Salt CLASSIC), 200 μg/ml ampicillin, 200 μg/ml streptomycin) at 28 °C with rotary shaking at 170 rpm. *Aurantiochytrium* sp. T66 (ATCC® PRA-276™) was cultured in YPDS (2% glucose, 2% peptone, 1% yeast extract, 1.75% ocean salt, 200 μg/ml ampicillin, 200 μg/ml streptomycin) at 25 °C with rotary shaking at 170 rpm (Supplemental Table S1). For plates, 20 g/L agar (LP0011, Oxoid Ltd, UK) was added. For long-term storage, cells were suspended in 15% glycerol and kept at −80 °C.

The fat accumulation medium was prepared as described: (in g/l) glucose (40), NH_4_Cl (0.7), KH_2_PO_4_ (0.3), Na_2_SO_4_ (18), MgSO_4_x7H_2_O (0.25), CaCl_2_x2H_2_O (0.2), KCl (0.4), Tris-base (6.1), maleic acid (5.8), and yeast extract (0.3). The pH of medium was adjusted to 7 with NaOH. Immediately before use, every liter of the medium was supplemented with 5 ml of trace mineral solution (in mg/l: 390 CuSO_4_x5H_2_O, 20 CoCl_2_x6H_2_O, 5000 FeSO_4_x7H_2_O, 150 MnSO_4_x7H_2_O, 10 NaMoO_4_x2H_2_O, and 440 ZnSO_4_x7H_2_O) and 1 ml of vitamin solution (in g/l: 0.05 thiamin HCl and 0.005 B12 cobalamin).

### Phylogenetic analysis

The analyses were conducted in MEGA X (Kumar et al. 2018) by using the maximum likelihood method and JTT matrix-based model (Jones et al. 1992). The analysis involved 31 amino acid sequences. There were a total of 546 positions in the final dataset. Initial trees for the heuristic search were obtained automatically by applying Neighbor-Join and BioNJ algorithms to a matrix of pairwise distances estimated using the JTT model and then selecting the topology with superior log likelihood value.

### Plasmid construction

PCRs were performed by Q5® High-Fidelity DNA Polymerase (New England Biolabs, USA). DNAs were digested by restriction enzymes (New England Biolabs, USA) and purified from gels by Monarch® DNA gel extraction kit (New England Biolabs, USA). Genomic DNAs were isolated by MasterPure™ Complete DNA and RNA Purification Kit (Lucigen, USA). Plasmids were extracted by ZR Plasmid Miniprep (Zymo Research, USA). DNAs were cloned into TOPO vectors by Zero Blunt™ TOPO™ PCR Cloning Kit (Invitrogen, USA). DNAs were ligated by T4 DNA Ligase (New England Biolabs, USA), followed by standard *E. coli* DH5α transformation and antibiotic selection. All PCR-originated regions of the clones are verified by Sanger sequencing (Eurofins Scientific, Luxembourg). Plasmids, primers, restriction enzymes, and antibiotic selection genes used were indicated in Supplemental Table S2, S3 and Fig. S1. Gene *T66des9* (T66002957.1) is the base pair position 4173-5426 of the T66 genome contig LNGJ01004217 (GenBank accession number) (Liu et al. 2016).

The scheme of the plasmid construction was illustrated in Supplemental Fig. S1. DNA fragment (r)*ble*-2A-*T66des9*-(f)GAPt was generated by overlap extension PCR. The 1st round PCR products A and C were amplified from pUC19-18GZG, and product B was amplified from *Aurantiochytrium* sp. T66 genomic DNA. PCR products A, B, and C were purified from gels and mixed as the 2nd round PCR template (Hilgarth and Lanigan 2020). DNA fragment GAPp-(f)*ble* and (r)GAPt were amplified from pUC19-18GZG. Upper and lower flanking were amplified from *A. limacinum* SR21 genomic DNA.

### Transformation of *A. limacinum* SR21

The electrotransformation protocol of *A. limacinum* SR21 was adapted from Faktorová et al. (2020) and Rius (2021). Cell colonies were inoculated in GPY medium at 28 °C for 2 days with rotary shaking at 170 rpm, followed by sub-culturing cells with starting OD_600_~0.02 in 50 ml GPY medium with 250 ml non-baffled flasks and 170 rpm rotary shaking at 28 °C until the OD_600_ reached around 3 (~24 h). Cells were then collected by centrifugation at 7,000 g for 5 min at 4 °C and washed by 10 ml of 1X BSS (10 mM KCl, 10 mM NaCl, and 3 mM CaCl_2_) twice by 10 ml of 50 mM sucrose with the same centrifugation setting and resuspended in 2 ml of 50 mM sucrose to a final concentration of approximately 7.5×10^5^ cells/μl. The cell suspensions were transferred to 2-mm-gap cuvettes (VWR, Belgium) on ice and adjusted to appropriate volumes (typically 150~300μl) with impedance (kΩ) between 0.9 and 1.5, measured by NEPA21 Electroporator (Nepa Gene Co., Ltd., Japan). DNA cassettes were linearized by *Sfo*I digestion at the plasmid backbone region and purified (Monarch® Kits for DNA Cleanup, New England Biolabs, USA). Ten μl DNA (3~5 μg) was added to the cell suspension in 2-mm-gap cuvettes, mixed by flicking, and incubated on ice for 5 min. Each cuvette was set on the NEPA21 Electroporator and pulsed with poring pulse parameters: 275 V, 8 milliseconds (ms) pulse length, two pulses, 50   ms length interval, 10% decay rate, “+” polarity and transfer pulse parameters: 20 V, 50 ms pulse length, 50 ms length interval, 1 pulse, 40% decay rate, “+/−” polarity. Two ml of GPYS medium was immediately added to the pulsed cells, which was then transferred to tubes and incubated overnight at 28 °C with rotary shaking at 170 rpm. The cells were collected by centrifugation and plated evenly to three GPYS agar plates (avoid over-dried) containing 100 μg/mL Zeocin (Thermo Fisher Scientific, USA) and incubated at room temperature for 3–5 days. Emerged colonies were re-streaked to new GPYS agar plates containing 100 μg/ml Zeocin. Colonies that grew after re-streaking were subjected to genomic DNA extraction (MasterPure™ Complete DNA and RNA Purification Kit, Lucigen, USA) and verified by PCR (Q5® High-Fidelity DNA Polymerase, New England Biolabs, USA).

### RT-PCR analysis

Total RNA from *A. limacinum* SR21 cells cultivated overnight in GPY medium were isolated using Spectrum™ Plant Total RNA Kit (Sigma-Aldrich, USA) with DNase I treatment (DNA-free™ DNA Removal Kit, Thermo Fisher Scientific, USA), and the cDNA was synthesized using First-Strand cDNA Synthesis Kit (Cytiva, USA) with random hexamer primers. PCR was performed to amplify parts of *T66des9* cDNA by the primer pair 2957RT-f and 2957RT-r, *ble* cDNA by the primer pair zeo sjekk-f and zeoRT-r, and β-tubulin gene (GenBank accession number: KX668278) cDNA as a reference gene by the primer pair SR21tubF2 and SR21tubR2 (Supplemental Table S3). PCR was performed by Q5® High-Fidelity DNA Polymerase (New England Biolabs, USA), with the parameters: 98 °C for 30 s, followed by 28 cycles of 98 °C for 10 s, 66 °C for 30 s, and 72 °C for 30 s, and followed by a final extension of 2 min at 72 °C.

### Cultivations for determination of fatty acid profiles

Single colonies of *A. limacinum* SR21 were inoculated in tubes with GPY medium at 28 °C for 2 days with rotary shaking at 170 rpm, followed by sub-culturing cells with starting OD_600_~0.02 in 100 ml fat accumulation medium with 500 ml baffled flasks and 170 rpm rotary shaking at 28 °C. For every collection time point, culture OD_600_ was recorded, and 2 ml of the culture was stored at −20 °C for FA analysis. For lipid extraction and determination of the FA isomers, 50 ml of the culture was centrifuged, and the pellet was stored at −20 °C until freeze-drying and analyses.

### Fatty acid analyses

The FA concentrations were determined by LC-MS (Heggeset et al. 2019). For the determination of the FA isomers, total lipids were extracted from the freeze-dried pellets (Jakobsen et al. 2008). The extracted oil was diluted with isooctane (to ca. 80 μg/μl) and methylated (AOCS 2017a; AOCS 2017b), before analysis on an Agilent 8860 gas chromatograph (Agilent Technologies, USA) equipped with a flame ionization detector and a CP-Wax 52 CB capillary column (25 m × 250 μm × 0.2 μm, Agilent Technologies, USA). The FAs were identified based on the GLC Reference standard 68D (GCL-68D, Nu-Check, USA), which contains 20 fatty acid methyl esters (FAMEs).

## Results

### Sequence analysis of *T66des9*

**Fig. 2:**
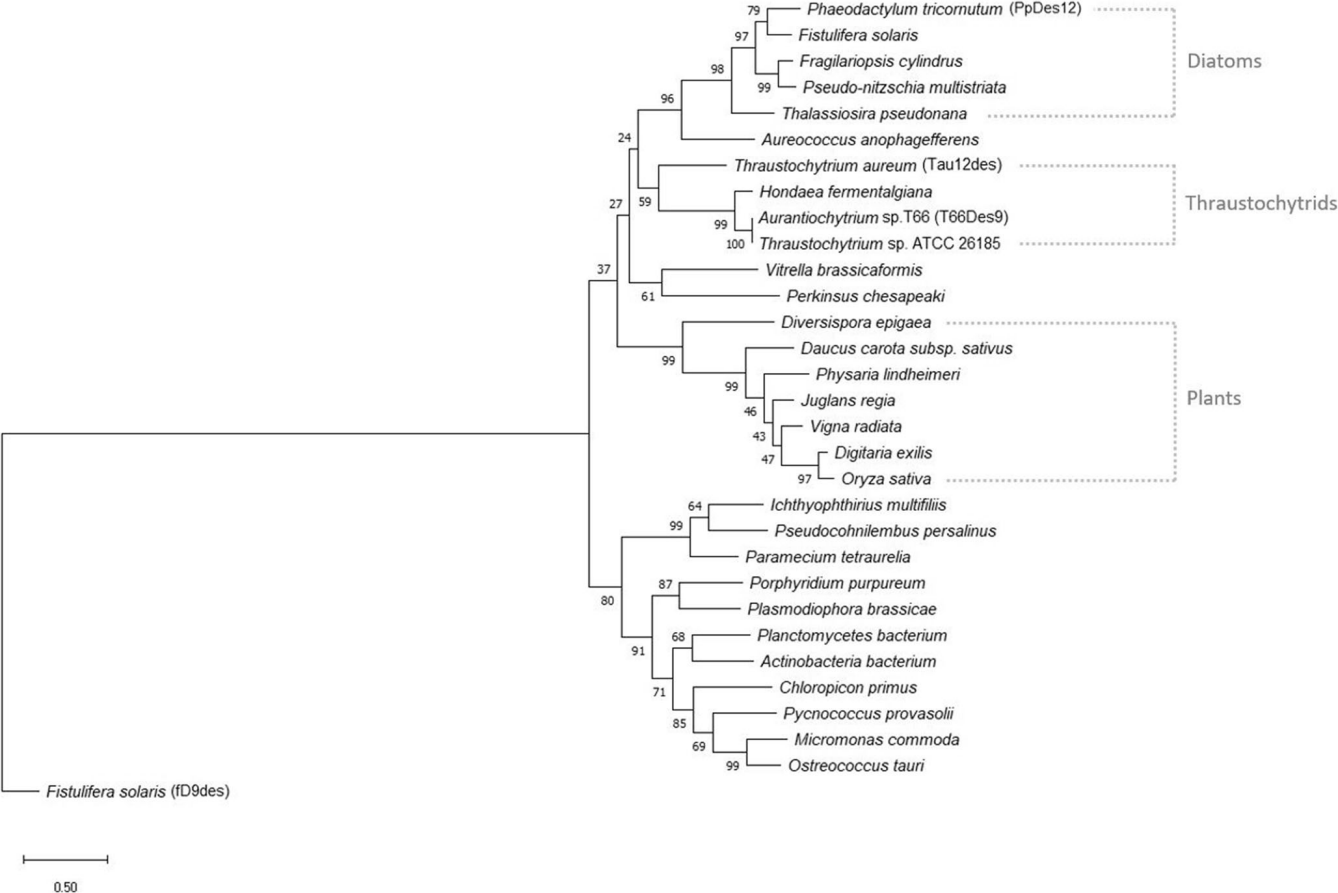
Phylogenetic analysis of T66Des9 and its homologs from the GenBank search. The homologs’ names and information were summarized in Supplemental Table S4 and shown here only with the species name unless selectively indicated with parentheses. The tree with the highest log likelihood (−20940.42) is shown. The tree is rooted with fD9des. The scale bar shows a distance of 0.5 substitutions per residue. The percentage of replicate trees in which the associated taxa clustered together in the bootstrap test (1000 replicates) are shown next to the branches (Felsenstein 1985)

**Fig. 3:**
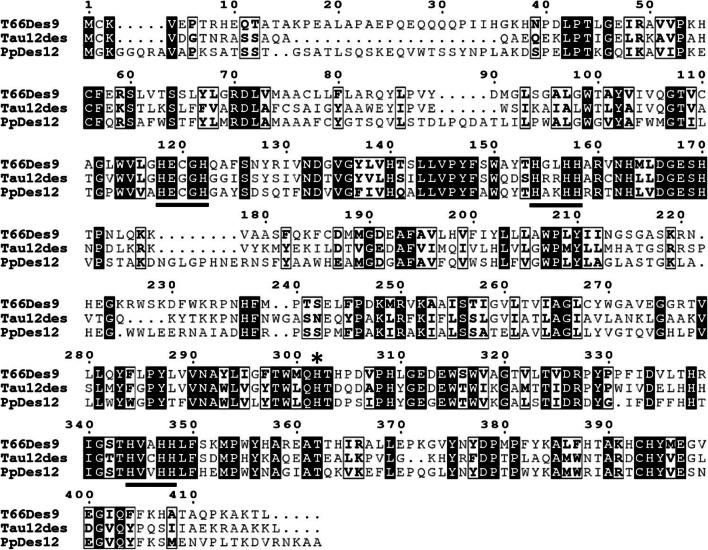
Multiple sequence alignment of the amino acid sequence of T66Des9 with the two most homologous proteins with characterized enzyme activity found in our search was generated by ClustalW and drawn by ESPript 3.0 (Robert and Gouet 2014). Tau12des: TauΔ12des, a Δ12-desaturase of *T. aureum*; PpDes12: a Δ12-desaturase of *P. tricornutum*; white letters with a black background: identical residues; bold letters in a black box: similar residues; underline: the three conserved histidine boxes of membrane-bound FA desaturases; asterisk: the conserved His302

We wanted to know if other species or strains encode homologs of T66Des9. The amino acid sequence of T66Des9 was first compared to the non-redundant protein sequences database at GenBank using BLAST. T66Des9 showed the highest degree of identity with proteins that are either annotated as Δ12-desaturase or hypothetical proteins, but no Δ9-desaturases (Supplemental Table S4). Phylogenetic analysis showed that T66Des9 is most closely related to proteins from other thraustochytrids, followed by diatoms and plants (Fig. 2). Two of the proteins most similar to T66Des9, TauΔ12des of *T. aureum* and PpDes12 of *Phaeodactylum tricornutum*, have both been verified to exhibit Δ12-desaturase activities (Domergue et al. 2003; Matsuda et al. 2012). To compare the sequence between T66Des9 and a functionally verified Δ9-desaturase, we added fD9des of *Fistulifera solaris* to the phylogenetic analysis (Muto et al. 2013). However, fD9des is substantially distant from the rest of the analyzed proteins, including a putative Δ12-desaturase from the same species. The structure of T66Des9 was then analyzed. Homology modeling using Phyre2 (Kelley et al. 2015) indicated that only the sequences of membrane-bound FA desaturases from mammals (Bai et al. 2015; Wang et al. 2015) were sufficiently similar to obtain a model. T66Des9 was found to have the four expected trans-membrane helixes (not shown). The protein contains the three histidine boxes with eight histidine residues and the single histidine residue after the last transmembrane helix (His302 in T66Des9), all of which are necessary for coordinating the di-iron molecule in the catalytic center (Nachtschatt et al. 2020; Nagao et al. 2019) (Fig. 3). The first histidine box has the consensus sequence HX_3_H, not the consensus sequence HX_4_H prevalent among Δ9-desaturases (Shanklin et al. 1994). We further compared the T66Des9 sequence to the *Labyrinthulomycetes* part of the whole genome shotgun database at NCBI and the sequenced *heterokonts* available at JGI. This identified a few additional thraustochytrid species encoding proteins closely related to T66Des9 (Supplemental Table S5). Notably, *A. limacinum* did not encode proteins closely related to T66Des9.

### Generation of a *T66des9*-expressing *A. limacinum* SR21 mutant strain

**Fig. 4:**
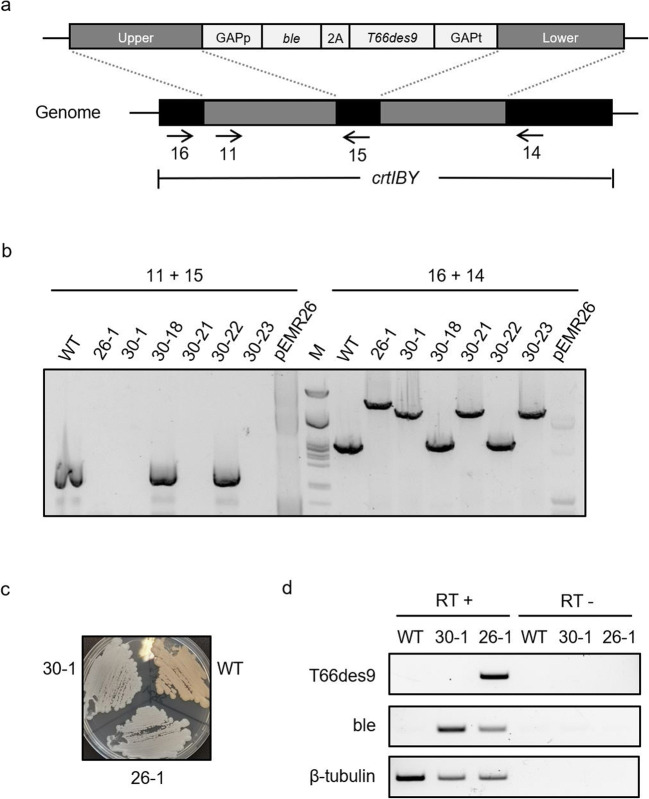
**a** Scheme for linearized pEMR24 or pEMR26 (without 2A and *T66des9*) cassettes integrating into the genome of *A. limacinum* SR21 cells. Upper/lower, homologous regions; GAPp/t, GAPDH promoter/terminator; *ble*, Zeocin resistance gene; 2A, peptide self-cleavage sequence; arrows, primer annealing sites. **b** Genomic PCRs of indicated strains were performed with the primer pairs 11+15 and 16 + 14. M: lambda-PstI ladder. **c** Indicated strains were streaked on GPYS agar medium and incubated at room temperature for about 2 weeks. **d** The transcripts of the indicated strains were detected by RT-PCR using specific primers for the *T66des9*, *ble*, and β-tubulin gene; RT+/-, template RNA with/without reverse transcription

It was decided to insert an expression cassette encoding T66Des9 into the *crtIBY* gene of *A. limacinum* SR21 by homologous recombination. The expression cassette contains a Zeocin resistance gene (*ble*) linked to *T66des9* by a 2A peptide-encoding DNA fragment, and gene expression was controlled by the endogenous glyceraldehyde 3-phosphate dehydrogenase (GAPDH) promoter and terminator (Fig. 4a and Supplemental Fig. S1). The 2A peptide can be cleaved off during protein translation, resulting in two separated proteins (Liu et al. 2017). The cassette was flanked by two DNA fragments from the β-carotene synthesis gene *crtIBY* (Iwasaka et al. 2018). Disruption of *crtIBY* will turn the colonies from brown to pale due to the lack of carotenoids, making this a convenient integration site since the site-specific genome integration does not affect viability or growth and can be verified by the color of the colonies (Rius 2021). The vector containing the *T66des9* knock-in cassette was designated pEMR24. Since the precursor of carotenoids is also acetyl-CoA, disruption of *crtIBY* could potentially affect FA synthesis. Moreover, the use of the endogenous GAPDH promoter might alter the expression of GAPDH and influence glycolysis, gluconeogenesis, and glycerol synthesis. Considering these potential side effects, we constructed plasmid pEMR26 to be able to create a control strain. pEMR26 is identical to pEMR24, except for it only encoding *ble* (Supplemental Fig. S1).

pEMR24 and pEMR26 were used to transform *A. limacinum* SR21 cells by electroporation. We obtained one colony (26-1) that could grow after re-streaking on a plate containing Zeocin after transformation by pEMR24 and five such colonies (30-1, 30-18, 30-21, 30-22, 30-23) after transformation by pEMR26. DNA from these colonies were then used in a PCR reaction that confirmed that 26-1 and three of the five transformants (30-1, 30-21, and 30-23) of pEMR26 had replaced parts of *crtIBY* with the plasmid inserts (Fig. 4a, b and Supplemental Fig. S2). Strains 26-1 and 30-1 encoding *ble-*2A*-T66des9* and *ble*, respectively, were chosen for further work. These strains also showed the expected pale phenotype which further supported that the expression cassette was correctly integrated (Fig. 4c).

While resistance to Zeocin indicated that the first part of the construct was expressed, we wanted to confirm that the *T66des9* gene transcripts were present in 26-1 strain. This was done by isolating RNA from the wild type (WT), strain 30-1 and 26-1, creating cDNA and then performing *T66des9* PCR. Samples without reverse transcriptase were used to rule out contamination of genomic DNA in the RNA samples. The results showed the presence of *T66des9* mRNA in 26-1 (Fig. 4d).

### Analyses of the fatty acid compositions of the T66Des9-expressing transgenic *A. limacinum* SR21 strains

Given that the gene was expressed, it was likely that the protein also would be expressed. In that case, analyses of the FAs synthesized by the mutant strain could demonstrate the function of T66Des9.

**Fig. 5:**
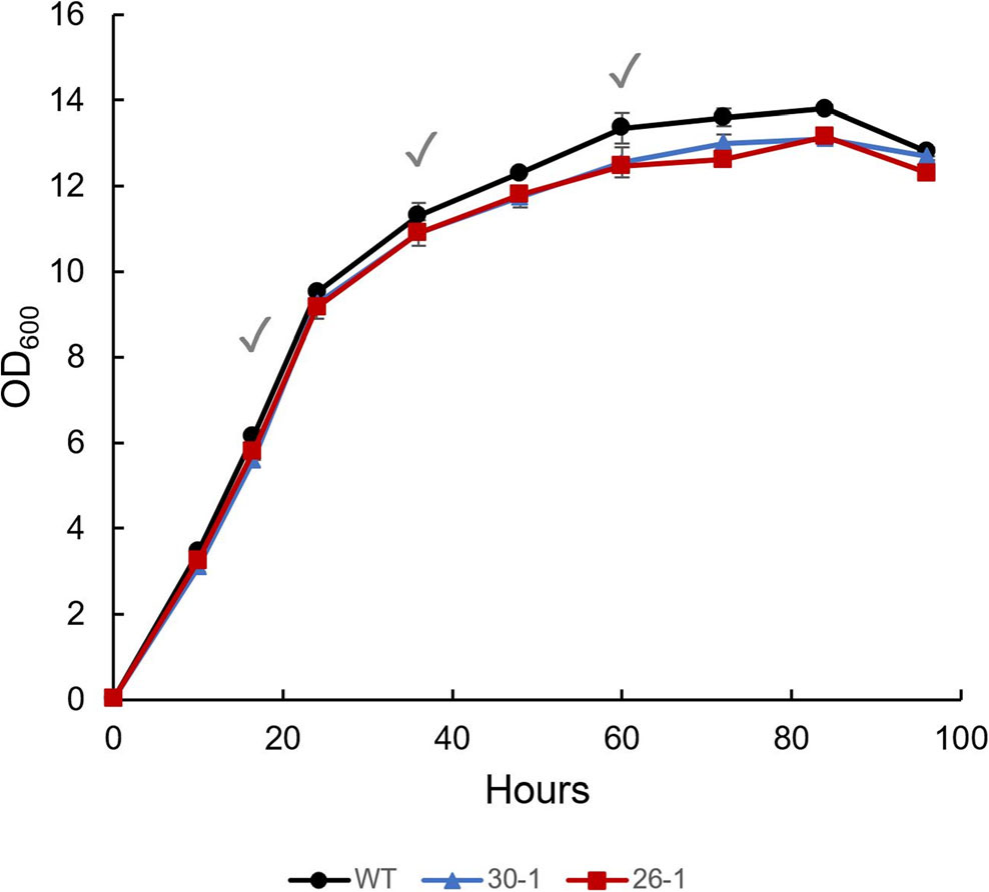
The growth of *A. limacinum* SR21 strain WT, 30-1 and 26-1 in shake flasks using glucose as the carbon source, measured as optical density (OD_600_); data are expressed as the mean of two replicates originating from two independent cultures; error bars represent the variation; checkmarks, sample collecting time points

*A. limacinum* SR21 WT and strains 30-1 and 26-1 were cultivated in shake flasks using glucose as the carbon source. The growth curves were similar for all three strains (Fig. 5). It should be mentioned that since the thraustochytrid cells accumulate lipids in the stationary phase, the optical density will still increase after they have stopped cell division. This becomes apparent in all studies plotting both optical density and lipid-free cell mass. We analyzed the FA composition of the strains after cultivating for 16.5 (approximately at nitrogen-exhaustion), 36 (nitrogen-limited), and 60 h (late nitrogen-limited). Supplemental Table S6 shows the data for all time points and Table 1 the data for the last sampling of the two transgenic strains. As expected, neither C16:1 nor C18:1 was detected in WT or strain 30-1 at any time point. On the other hand, both C16:1 and C18:1 were detected in strain 26-1.

**Table 1:** Fatty acid composition of *A. limacinum* SR21 strains after cultivation for 60 h. (Data are expressed as the mean ± the variation of two replicates originating from two independent cultures. The data for each replicate are the mean of two separate runs of FA analysis. The FAs less than 0.01 g/l were considered as background signals and were not shown)

Strains	30-1		26-1	
FAs	g/l^a^	%^b^	g/l	%
C14:0	0.36±0.01	4.62±0.44	0.38±0.04	5.07±0.36
C16:0	4.35±0.3	55.18±0.92	3.99±0.19	52.94±0.02
C16:1	0.00	0.00	0.2±0.01	2.66±0.07
C18:0	0.09±0.04	1.1±0.54	0.1±0	1.4±0.09
C18:1	0.00	0.00	0.18±0	2.37±0.14
C20:5	0.02±0	0.3±0.01	0.02±0	0.29±0.02
C22:5	0.77±0.04	9.66±0.74	0.71±0.03	9.42±0.86
C22:6	2.31±0.18	29.11±0.82	2.05±0.14	27.13±0.57
Total	6.87±1.85		7.54±0.35	

^a^Grams per liter of the culture

^b^Percentage of total fatty acids

**Fig. 6:**
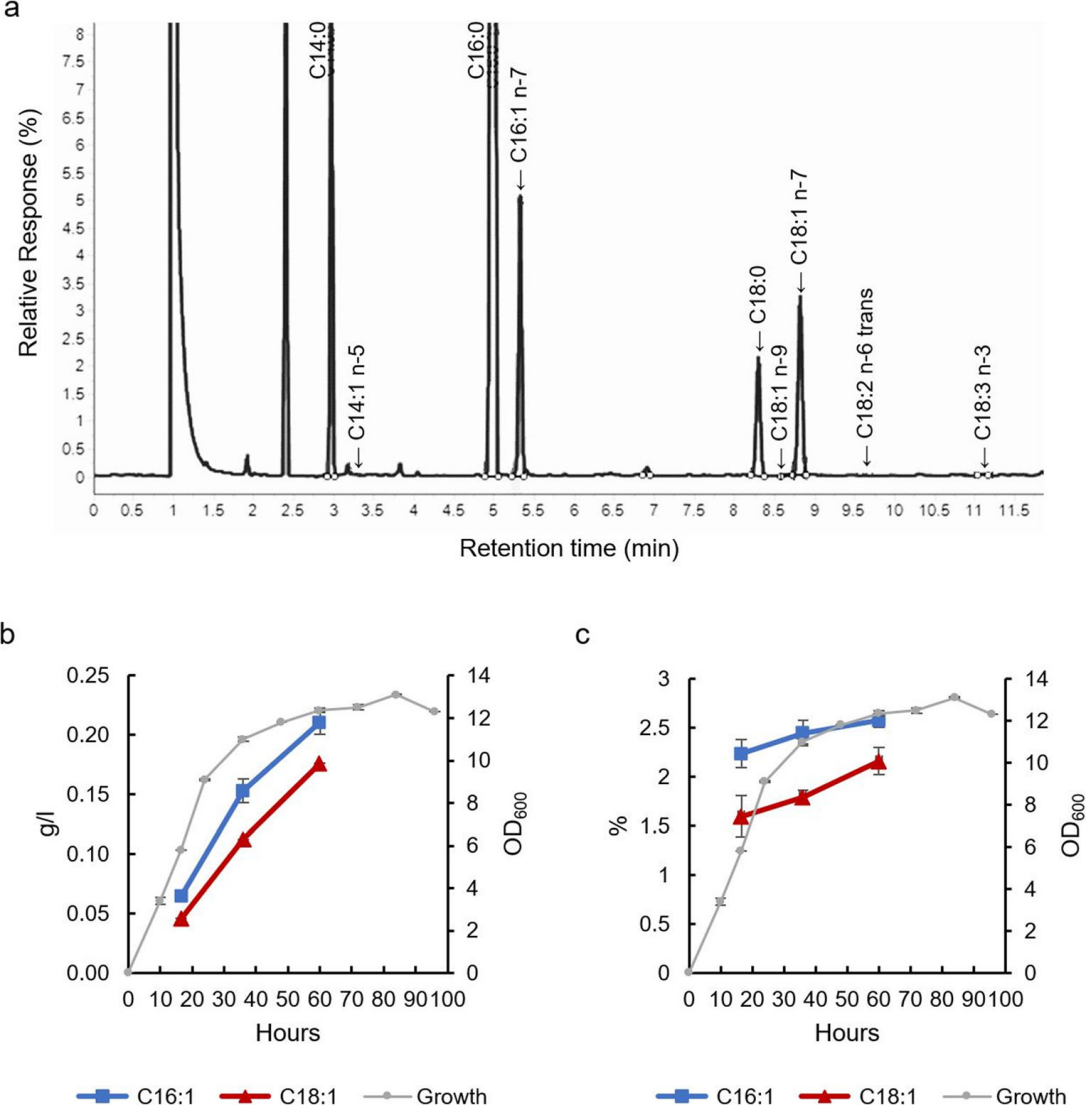
**a** Partial gas chromatography chromatogram of FAMEs from *A. limacinum* SR21 strain 26-1 cultured for 60 hours. The positions of the FAs were based on the standard GCL-68D. The time course accumulation of C16:1 and C18:1 from *A. limacinum* SR21 strain 26-1 is shown by grams per liter of culture (**b**) and the percentage of total fatty acids (**c**). Data are the mean of two replicates originating from two independent cultures. The data for each replicate are the mean of two separate runs of FA analysis; cell growth is the mean of two replicates originating from two independent cultures and indicated as OD_600_; error bars represent the variation

In order to determine the position of the double bond, methyl esters of the FAs produced by strain 26-1 and *Aurantiochytrium* sp. T66 WT as control were analyzed by GC-FID. The results showed that both C16:1 and C18:1 were the n-7 isomers (Fig. 6a and Supplemental Table S7). This indicated that T66Des9 is a Δ9-desaturase when C16:0 is the substrate.

## Discussion

In the present study, expression of *T66Des9* resulted in the synthesis of C16:1 n-7 and C18:1 n-7 in *A. limacinum* SR21. Since the amount of C18:0 was lower than that of C18:1 n-7 and the amount of C16:1 n-7 and C18:1 n-7 accumulated nearly linearly at similar rates (Fig. 6 b and c), it is likely that C18:1 n-7 was synthesized from C16:1 n-7 by an elongase (Fig. 1). In *Aurantiochytrium* sp. T66, the putative protein encoded by T66003689.1 (ELO-1) is identical to the function-verified C16-Δ9 elongase TsELO2 from *Thraustochytrium* sp. ATCC 26185 (Heggeset et al. 2019; Ohara et al. 2013). The protein encoded by Aurli1_73494 of *A. limacinum* SR21 shows 72% identity to TsELO2, and this protein or other putative elongases might be able to accept C16:1 n-7 as substrate.

Our data cannot exclude the possibility that the enzyme T66Des9 is an ω-7 desaturase, counting from the methyl end instead of the carboxyl end indicated by the Δ-nomenclature. Still, we have not found any reports in literature on ω-type enzymes introducing the first double bond. Some plants also make n-7 fatty acids, and it has been shown that they do so by using Δ9 desaturases (see, e.g., Cahoon et al. 1998). However, since the plant enzymes utilize ACP-linked and not CoA-linked substrates, this merely is an indication. Taken together, the most likely interpretation of our data is that T66Des9 is a Δ9-desaturase that accepts C16:0 as a substrate.

Phylogenetic studies of desaturases have shown that Δ9-desaturases and Δ12-desaturases are well separated (Sperling et al. 2003; Wilding et al. 2017). However, the same papers also point out that since the assumed gene duplication and subsequent functional change to Δ9-desaturases or Δ12-desaturases took place fairly early in evolution, much of the amino acids now found to be specific for Δ9-desaturases or Δ12-desaturases might not really be necessary for their specific functions. Still, Fig. 1 shows that T66Des9 belongs to the clade identified as Δ12-desaturases (Wilding et al. 2017). A possible explanation could be that the Δ12-desaturase gene in an ancestral thraustochytrid cell became duplicated. Then, one of the duplicate genes evolved, possibly through the step of encoding a bifunctional Δ9/Δ12-desaturase into a Δ9-desaturase gene. In the ancestral T66 cell, the Δ12-desaturase gene was then lost. One similar incident has been described earlier in the house cricket (*Acheta domesticus*) (Zhou et al. 2008). This insect encodes a bifunctional Δ12/Δ9-desaturase AdD9des with low Δ9 desaturation activity that presumably evolved from a Δ9-desaturase (Zhou et al. 2008). The Δ9 desaturation activity of AdD9des could also be enhanced by point mutations, identified via directed evolution approaches, indicating that the Δ12 regioselectivity of AdD9des could gradually have been obtained by an ancestral Δ9-desaturase (Vanhercke et al. 2011). No di-unsaturated FA were identified in our experiments, indicating that T66Des9 is not a bifunctional enzyme.

One of the reasons that thraustochytrids may not usually generate DHA through the DE pathway is that many strains are missing one or more of the expected DE pathway desaturases and elongases (Dellero et al. 2018; Liang et al. 2020; Meesapyodsuk and Qiu 2016). For example, here we demonstrated that *Aurantiochytrium* sp. T66 lacks the Δ12-desaturase annotated previously, because the gene instead encodes the Δ9-desaturase T66Des9 synthesizing C16:1 n-7, a FA that is not considered as a DHA precursor. Our findings are consistent with the possibility described in previous reports that *Aurantiochytrium* sp. T66 does not possess an intact FAS-DE pathway to produce DHA (Heggeset et al. 2019; Jakobsen et al. 2008).

C16:1 n-7 and a non-oleic C18:1 FA can be produced in *Thraustochytrium* sp. ATCC 26185 (Weete et al. 1997). It has earlier been demonstrated that C18:1 n-7 can be synthesized from C16:1 n-7 by TsELO2 of this strain (Ohara et al. 2013), while it was not known which enzyme produces C16:1 n-7. Since *Thraustochytrium* sp. ATCC 26185 has an identical protein to T66Des9, our discovery seems to be fitting the last piece into the puzzle of how *Thraustochytrium* sp. ATCC 26185 can produce the C18:1 n-7, presumably the non-oleic C18:1 found in the strain. Conjugated linoleic acid, the downstream product of C18:1 n-7, is substantially beneficial to human metabolism (Koba and Yanagita 2014). The potential values of C18:1 n-7 itself on health are also expanding (Blewett et al. 2009; Jacome-Sosa et al. 2016). The knowledge gained from this study demonstrates the potential of utilizing thraustochytrids to produce C18:1 n-7 by metabolic strain engineering.

## Supplementary Information

ESM 1 (PDF 210 kb)

## Data Availability

The strains developed in this study are available upon request. All data generated or analyzed during this study are included in this published article and its supplementary information files.
