# Supplementary material for: A non-canonical Δ9-desaturase synthesizing palmitoleic acid identified in the thraustochytrid Aurantiochytrium sp. T66
Source: Appl Microbiol Biotechnol. 2021 Jul 22;105(14-15):5931–41. doi: 10.1007/s00253-021-11425-5 (PMC8390409; doi:10.1007/s00253-021-11425-5)
Supplement: Supplementary file 1 — (PDF 210 kb) [file 253_2021_11425_MOESM1_ESM.pdf]

Supplementary Information

Applied Microbiology and Biotechnology

A non-canonical Δ9-desaturase synthesizing palmitoleic acid identified in the thraustochytrid *Aurantiochytrium* sp. T66

Authors

E-Ming Rau[1], Inga Marie Aasen[2], Helga Ertesvåg[1*]

[1]Department of Biotechnology and Food Science, NTNU Norwegian University of Science and Technology, Trondheim, Norway

[2]Department of Biotechnology and Nanomedicine, SINTEF Industry, Trondheim, Norway

* Correspondence to: helga.ertesvag@ntnu.no

Supplemental Table S1 Strains used in this study

Strain	Description	Reference
T66	Wild-type *Aurantiochytrium* sp. T66	ATCC®
SR21	Wild-type *Aurantiochytrium limacinum* SR21	ATCC®
#26-1	Transgenic strain derived from SR21 with *crtIBY* disrupted by *ble*-2A-*T66des9* expression cassette	this study
#30-1	Transgenic strain derived from SR21 with *crtIBY* disrupted by *ble* expression cassette	this study

Supplemental Table S2 Plasmids used in this study

Plasmids	Description	Reference
pUC19-18GZG	Amp^R, *ble* flanked by SR21 endogenous GAPDH promoter and terminator	Addgene
pEMR15	Kan^R, pCR™Blunt II-TOPO® containing *ble* (rear part)-2A-*T66des9*-GAPDH terminator (front part)	This study
pEMR19	Kan^R, pCR™Blunt II-TOPO® containing *crtIBY* homologous flanking sequence (upper)	This study
pEMR20	Kan^R, pCR™Blunt II-TOPO® containing GAPDH promoter-*Ble* (front part)	This study
pEMR21	Kan^R, pCR™Blunt II-TOPO® containing GAPDH terminator (rear part)	This study
pEMR22	Kan^R, pCR™Blunt II-TOPO® containing *crtIBY* homologous flanking sequence (lower)	This study
pEMR23	Amp^R, containing GAPDH terminator (rear part)-*crtIBY* homologous flanking sequence (lower)	This study
pEMR24	Amp^R, GAPDH promoter-*ble*-2A-*T66des9*-GAPDH terminator expression cassette flanked by *crtIBY* homologous sequences	This study
pEMR26	Amp^R, GAPDH promoter-*ble*-GAPDH terminator expression cassette flanked by *crtIBY* homologous sequences	This study

Supplemental Table S3 Primers used in this study

Primer short name	Primer	Sequence 5'→3'	Underlined region
1	KI2957-1-SmaI	CTCCCGGGACTTCGTG	*Xma*I/*Sma*I
2	KI2957-2-2a	GTCTCCTGCTTGCTTGAGCAGAGAGAAGTTCGTGGCTCCGGATCCGTCCTGCTCCTCGGC	partial 2A sequence
3	KI2957-3-2a	GCCACGAACTTCTCTCTGCTCAAGCAAGCAGGAGACGTGGAAGAAAACCCCGGTCCTTGCAAGGTGGAGCCC	partial 2A sequence
4	KI2957-4	TACATGTCGACTCTAGAGGATCCCCTTAGAGCGTCTTGGCCTTG	
5	KI2957-5	GGGGATCCTCTAGAGTCGAC	
6	KI2957-6	CAGAAATACTAGTTTTGTGAATGAAAAGAGATGATAAAAG	*Spe*I
7	GAPDHpro_SbfI-F	ATACCTGCAGGGAGACGAGCATATGACTACTG	*Sbf*I
8	Ble_inside_SmaI-R	ACGAAGTCCCGGGAGAAC	*Xma*I/*Sma*I
9	GAPDHter_inside-SpeI-F	CATTCACAAAACTAGTATTTCTGCATTAGAAATC	*Spe*I
10	GAPDHter-NotI-R	ATAGCGGCCGCAGCGGATAACAATTTCACACAGG	*Not*I
11	SR21 CrtUF	GGTACCTGGATGCCTGAGGTCTTC	*Kpn*I
12	SR21 CrtUR	GGGCCCATTAATCCTGCAGGGAATACATGGCTGCGCTAC	*Sbf*I
13	SR21 CrtD-NotI-F	ATAGCGGCCGCCGCTCGCTTTCTTGGATACTG	*Not*I
14	SR21 CrtDR	ATTGTCACAGGGCGAACG	
15	21crt-midR	CGGCCGTGTTCATATAAGAG	
16	21CrtUFcheck	TGGCAGAGCTCATCAGTTTG	
	zeo sjekk-f	AGTTGACCAGTGCCGTTCC	
	zeoRT-r	CGAAGTCGTCCTCCACGAAG	
	SR21tubF2	TGTTGAGAACGCTGATGAGG	
	SR21tubR2	CGAGCTTACGGAGGTCAGAG	
	2957RT-f	GTCAACGCCTACCTCATTGGG	
	2957RT-r	CTTGGAGAAGAGGTGGTGCG	

Supplemental Table S4 T66Des9 homologs that showed the highest degree of identity with proteins encoded by different genera or thraustochytrids species using BLAST in the non-redundant protein sequences database at Genbank

**Accession**	**Description**	**Species**	**Query Cover**	**E value**	**Per. Ident**
AOG21009.1	delta12 desaturase	*Thraustochytrium* sp. ATCC 26185	100%	0	100.00%
GBG24140.1	Delta12-fatty-acid desaturase	*Hondaea fermentalgiana*	100%	0	80.65%
BAM37464.1	delta12-fatty acid desaturase (TauΔ12des)	*Thraustochytrium aureum*	99%	1.00E-124	44.12%
AAO23564.1	delta 12 fatty acid desaturase (PpDes12)	*Phaeodactylum tricornutum*	97%	8.00E-119	44.34%
BAO27791.1	delta12 desaturase-b	*Fistulifera solaris*	90%	1.00E-110	44.19%
OEU07512.1	delta 12 fatty acid desaturase	*Fragilariopsis cylindrus* CCMP1102	91%	2.00E-109	44.25%
XP_002292071.1	predicted protein	*Thalassiosira pseudonana* CCMP1335	91%	3.00E-106	41.67%
VEU44279.1	unnamed protein product	*Pseudo-nitzschia multistriata*	90%	2.00E-103	42.57%
CEM11741.1	unnamed protein product	*Vitrella brassicaformis* CCMP3155	88%	1.00E-101	43.32%
XP_009035621.1	hypothetical protein AURANDRAFT_24467	*Aureococcus anophagefferens*	87%	2.00E-100	42.01%
MBC8351339.1	fatty acid desaturase	*Planctomycetes bacterium*	86%	7.00E-93	42.12%
KAA8495721.1	Omega-6 fatty acid desaturase, endoplasmic reticulum isozyme 2	*Porphyridium purpureum*	96%	8.00E-93	38.69%
NLA37757.1	fatty acid desaturase	*Actinobacteria bacterium*	85%	7.00E-87	41.32%
XP_002507091.1	fatty acid desaturase	*Micromonas commoda*	94%	6.00E-85	38.06%
CEO99628.1	hypothetical protein PBRA_007361	*Plasmodiophora brassicae*	90%	7.00E-84	37.99%
XP_001454547.1	hypothetical protein	*Paramecium tetraurelia* strain d4-2	85%	2.00E-83	39.28%
NLD75814.1	fatty acid desaturase	*Acidimicrobiales bacterium*	92%	2.00E-82	39.19%
HBU37186.1	TPA: fatty acid desaturase	*Planctomycetaceae bacterium*	91%	8.00E-82	37.98%
KAF4668261.1	linoleoyl-CoA desaturase activity	*Perkinsus chesapeaki*	87%	2.00E-81	38.92%
XP_003075374.1	Fatty acid desaturase, type 1	*Ostreococcus tauri*	86%	1.00E-80	38.21%
RHZ75025.1	hypothetical protein Glove_218g3	*Diversispora epigaea*	95%	6.00E-80	34.91%

QDZ17765.1	delta(12)-fatty-acid desaturase	*Chloropicon primus*	84%	2.00E-78	38.72%
ABQ01458.1	oleate 12-hydroxylase	*Physaria lindheimeri*	84%	2.00E-78	38.14%
XP_018860212.1	delta(12)-fatty-acid desaturase FAD2-like	English walnut	93%	3.00E-78	38.78%
XP_004025050.1	hypothetical protein IMG5_193510	*Ichthyophthirius multifiliis*	91%	6.00E-78	37.28%
KRX08182.1	hypothetical protein PPERSA_12337	*Pseudocohnilembus persalinus*	88%	7.00E-78	36.19%
GHP07573.1	linoleoyl-CoA desaturase	*Pycnococcus provasolii*	84%	9.00E-78	40.77%
XP_022640459.1	omega-6 fatty acid desaturase, endoplasmic reticulum isozyme 2 isoform X2	mung bean	91%	2.00E-77	39.16%
KAF8643095.1	hypothetical protein HU200_066967	*Digitaria exilis*	95%	2.00E-77	38.00%
KAB8088627.1	hypothetical protein EE612_013286	rice	89%	3.00E-77	38.96%
XP_017220518.1	PREDICTED: delta(12)-fatty-acid desaturase FAD2-like	Daucus carota subsp. sativus	90%	4.00E-77	37.86%

Supplemental Table S5 List of thraustochytrid species encoding proteins closely related to T66Des9 by using BLAST in the databases[a] at Genbank

	nr[b]		wgs[c]	
**Species**	**Accession**	**Identity (%)**	**Accession**	**Identity (%)**
Aurantiochytrium sp. KH105			BGKB01000047.1	87
Aurantiochytrium sp. KH105			BGKB01000033.1	80
Hondaea fermentalgiana strain FCC1311	GBG24140.1	80.65	NPFB01000004.1	81
Schizochytrium sp. CCTCC M209059			JTFK01001013.1	86
Thraustochytrium aureum ATCC 34304	BAM37464.1	44.12	BLSG01000191.1	43
Thraustochytrium sp. ATCC 26185	AOG21009.1	100		

[a] No relevant results were obtained from the sequenced *heterokonts* available at JGI database. [b] The non-redundant protein sequences database at Genbank. [c] The *Labyrinthulomycetes* part of the whole genome shotgun database at Genbank

Supplemental Table S6 Fatty acid compositions of *A. limacinum* SR21 strains: all analyzed timepoints [a]

	g/l[b]								
**Strain**	**WT**			**30-1**			**26-1**		
**Hr** / **FA**	16.5	36	60	16.5	36	60	16.5	36	60
C14:0	0.11±0.01	0.28±0.01	0.34±0.01	0.11±0.01	0.31±0.01	0.36±0.01	0.13±0.01	0.34±0.03	0.38±0.04
C15:0	0	0	0	0	0	0	0	0	0
C16:0	1.53±0.06	3.46±0.1	4.71±0.3	1.36±0.12	3.36±0.21	4.35±0.3	1.37±0.23	3.32±0.07	3.99±0.19
C16:1	0	0	0	0	0	0	0.06±0	0.15±0.01	0.2±0.01
C17:0	0	0	0	0	0	0	0	0	0
C18:0	0.02±0.03	0.05±0.07	0.12±0	0.04±0	0.06±0.03	0.09±0.04	0.04±0	0.09±0	0.1±0
C18:1	0	0	0	0	0	0	0.05±0	0.12±0	0.18±0
C20:5	0.02±0	0.02±0	0	0.01±0	0.02±0	0.02±0	0.01±0	0.02±0	0.02±0
C22:5	0.24±0.04	0.58±0.06	0.79±0.08	0.21±0	0.6±0.02	0.77±0.04	0.21±0.01	0.58±0.05	0.71±0.03
C22:6	0.74±0.11	1.8±0.12	2.63±0.48	0.64±0.02	1.78±0.01	2.31±0.18	0.66±0.08	1.66±0.02	2.05±0.14
**Total**	2.64±0.2	6.18±0.22	8.61±0.72	2.37±0.15	5.32±1.28	6.87±1.85	2.51±0.34	6.21±0.05	7.54±0.35

	%[c]								
**Strain**	**WT**			**30-1**			**26-1**		
**Hr** / **FA**	16.5	36	60	16.5	36	60	16.5	36	60
C14:0	4.18±0.17	4.48±0.26	3.93±0.23	4.51±0.02	5.11±0.22	4.62±0.44	5.01±0.15	5.47±0.45	5.07±0.36
C15:0	0	0	0	0	0	0	0	0	0
C16:0	58.04±2.27	55.98±0.32	54.72±1.02	57.43±1.47	54.81±2.13	55.18±0.92	54.69±1.63	53.51±0.66	52.94±0.02
C16:1	0	0	0	0	0	0	2.36±0.14	2.42±0.13	2.66±0.07
C17:0	0	0	0	0	0	0	0	0	0
C18:0	0.78±1.1	0.74±1.04	1.36±0.06	1.78±0.1	1.03±0.51	1.1±0.54	1.71±0.14	1.44±0.02	1.4±0.09
C18:1	0	0	0	0	0	0	2.08±0.21	1.89±0.07	2.37±0.14
C20:5	0.29±0.01	0.26±0.01	0	0.3±0.01	0.27±0.02	0.3±0.01	0.28±0.01	0.26±0.01	0.29±0.02
C22:5	8.89±0.99	9.37±1.38	9.19±1.65	8.69±0.58	9.75±0.61	9.66±0.74	8.52±0.61	9.31±0.82	9.42±0.86
C22:6	27.82±2.2	29.18±0.93	30.47±3.07	27.26±0.79	29±0.75	29.11±0.82	26.57±0.5	26.73±0.45	27.13±0.57

[a]Data are expressed as the mean ±variation of two replicates originated from two independent cultures. The data of each replicate of strain 30-1 and 26-1 are the mean of two separate runs of FA analysis. [b]Grams per liter of the culture. [c]Percentage of total fatty acids. The FAs less than 0.01 g/l were considered as background signals and were not shown

Supplemental Table S7 Fatty acid compositions of *A. limacinum* SR21 strain 26-1 and *Aurantiochytrium* sp. T66 WT with the isomers distinguished

	Peak Area % of identified FAMEs	
**Fatty acid**	**26-1**	**T66**
C14:0	3.76	13.75
C14:1 n-5	< 0.1	< 0.1
C16:0	55.67	32.82
C16:1 n-7	2.64	13.62
C18:0	1.57	1.23
C18:1 n-9	< 0.1	< 0.1
C18:1 n-7	2.45	8.17
C18:3 n-3	< 0.1	< 0.1
C20:0	0.20	< 0.1
C20:4 n-6	0.23	0.15
C20:5 n-3 (EPA)	0.26	0.48
C22:0	0.11	< 0.1
C22:5 n-3 (DPA)	5.99	7.97
C24:0	< 0.1	0.17
C22:6 n-3 (DHA)	26.99	21.42
Sum identified FAMEs (%)	99.40	98.19
Sum unknown peaks (%)	0.60	1.65
LOQ 0.1%		

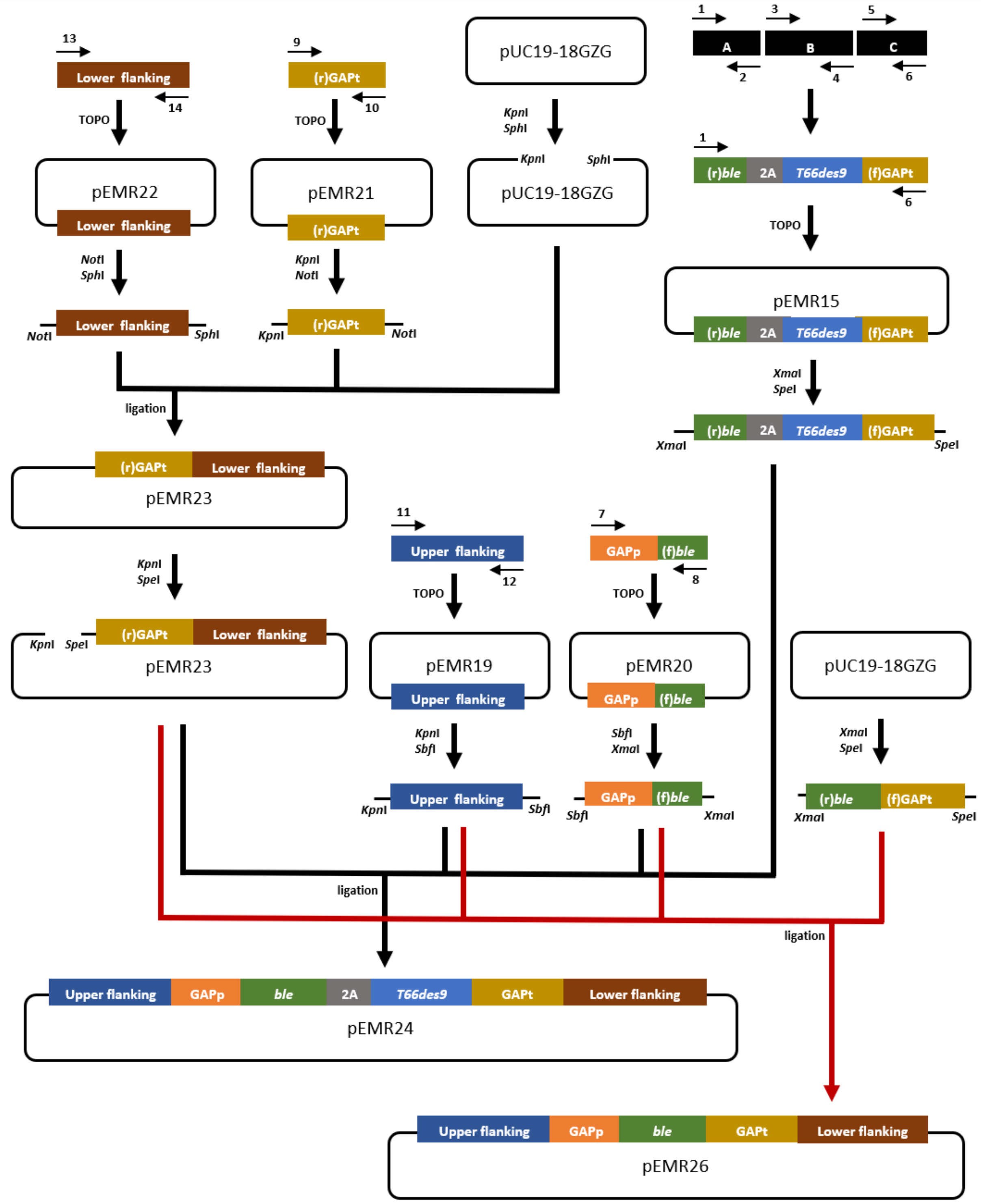

Supplemental Fig. S1 Scheme of constructing pEMR24 and pEMR26. Upper/Lower flanking: *crtIBY* homologous regions; GAPp: GAPDH promoter; GAPt: GAPDH terminator; (f)/(r)GAPt: the front/rear of GAPt ; *ble*: Zeocin resistance gene; (f)/(r)*ble*: the front/rear of *ble*; 2A: peptide self-cleavage sequence; 1~12: PCR primers

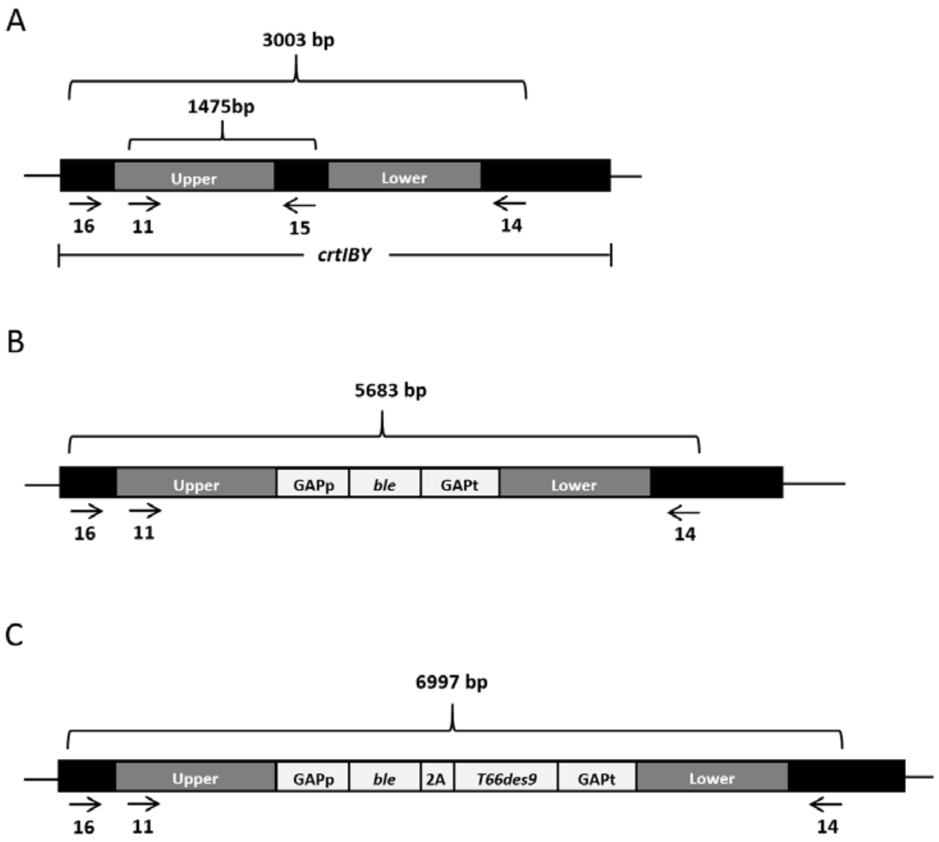

Supplemental Fig. S2 Illustration of the sizes of PCR products amplified from the gnomic DNA of (A) WT and non-transformants (30-18 and 30-22), (B) strain 30-1, 30-21 and 30-23, (C) strain 26-1. Primer pair 11+15 amplified fragments of 1475bp only from WT, 30-18 and 30-22. Primer pair 16 + 14 amplified larger PCR fragments of 6997bp and 5683bp from 26-1, and the three strains (30-1, 30-21 and 30-23), respectively. Primer pair 16 and 14 amplified a shorter fragment of 3003bp from WT, 30-18 and 30-22. Upper/Lower: *crtIBY* homologous regions; GAPp/t: GAPDH promoter/terminator; *ble*: Zeocin resistance gene; 2A: peptide self-cleavage sequence; Arrows: primer annealing sites
